# *EDN1-AS*, A Novel Long Non-coding RNA Regulating Endothelin-1 in Human Proximal Tubule Cells

**DOI:** 10.3389/fphys.2020.00209

**Published:** 2020-03-13

**Authors:** Lauren G. Douma, Kristen Solocinski, Sarah H. Masten, Dominique H. Barral, Sarah J. Barilovits, Lauren A. Jeffers, Kareme D. Alder, Ravi Patel, Charles S. Wingo, Kevin D. Brown, Brian D. Cain, Michelle L. Gumz

**Affiliations:** ^1^Department of Medicine, University of Florida, Gainesville, FL, United States; ^2^Department of Biochemistry and Molecular Biology, University of Florida, Gainesville, FL, United States; ^3^Department of Biochemistry, Cell and Developmental Biology, Emory University, Atlanta, GA, United States; ^4^Yale University School of Medicine, New Haven, CT, United States

**Keywords:** kidney, long non-coding RNA, proximal tubule cells, CRISPR, circadian rhythm

## Abstract

Endothelin-1 (ET-1) is a peptide hormone that functions as a vasoconstrictor in the vasculature, whereas in the collecting duct of the kidney it exerts blood pressure-lowering effects via natriuretic actions. Aberrant ET-1 signaling is associated with several pathological states including hypertension and chronic kidney disease. ET-1 expression is regulated largely through transcriptional control of the gene that encodes ET-1, *EDN1*. Here we report a long, non-coding RNA (lncRNA) that appears to be antisense to the *EDN1* gene, called *EDN1-AS*. Because *EDN1-AS* represents a potential novel mechanism to regulate ET-1 expression, we examined the regulation of *EDN1-AS* expression and action. A putative glucocorticoid receptor response (GR) element upstream of the predicted *EDN1-AS* transcription start site was identified using the ENCODE database and the UCSC genome browser. Two homozygous deletion clones of the element were generated using CRISPR/Cas9. This deletion resulted in a significant increase in the expression of *EDN1-AS*, which was associated with increased secretion of ET-1 peptide from HK-2 cells (two-fold increase in KO cells vs. CNTL, *n* = 7, *P* < 0.05). Phenotypic characterization of these CRISPR clones revealed a difference in cell growth rates. Using a standard growth assay, we determined that the KO1 clone exhibited a three-fold increase in growth over 8 days compared to control cells (*n* = 4, *P* < 0.01) and the KO2 clone exhibited a two-fold increase (*n* = 4, *P* < 0.01). These results support a role for *EDN1-AS* as a novel regulatory mechanism of ET-1 expression and cellular proliferation.

## Introduction

ET-1 has many functions including vascular constriction, nervous system activation, and renal sodium secretion. The kidney is both a source and a target of ET-1, and the kidney has the highest concentrations of ET-1 found in the body (Kohan et al., 2011). The mechanisms of ET-1 action in the pathology of chronic kidney disease (CKD) involve increased cell proliferation, inflammation, and elaboration of the extracellular matrix (Speed and Pollock, 2013; Kohan and Barton, 2014; Reichetzeder et al., 2014). Mice overexpressing ET-1 developed chronic renal failure (Theuring et al., 1998). In humans, excess ET-1 levels are seen in all stages of CKD (Dhaun et al., 2009), and there are marked increases in urinary ET-1 secretion in CKD (Grenda et al., 2007). ET-1 signaling works through two receptors, ETAR and ETBR, via paracrine and autocrine action (Dhaun and Webb, 2019). ETAR and ETBR exert largely opposite effects, and ET-1 stimulation of ETAR is primarily responsible for renal injury. Although regulation of ET-1 levels is widely attributed to control of transcription of the ET-1 gene (*EDN1*) (Stow et al., 2011), *EDN1* mRNA is also regulated at the post-transcriptional level by miRNAs (Jacobs et al., 2013, 2014).

The ET-1 pathway is a therapeutic target for many diseases. The ET receptor blocker Macitentan improved morbidity and mortality in pulmonary arterial hypertension patients (Pulido et al., 2013) whereas studies of ET-1 blockers in the kidney have been less successful. The endothelin axis is an important target in CKD, but pharmacological manipulation of endothelin receptors is associated with adverse side effects that have led to termination of clinical trials (Kohan and Pollock, 2013; Yuan et al., 2015). The ASCEND trial using ET-1 receptor blockers for CKD therapy was abandoned due to increased incidence of congestive heart failure (Reichetzeder et al., 2014). More recently, promising results emerged from SONAR, a trial for the ETA antagonist Atrasentan, which utilized an enrichment protocol to mitigate fluid retention side effects (Heerspink et al., 2019). Atrasentan reduced the risk for renal events in patients with type 2 diabetes mellitus, although the trial was ended early due to a less than expected number of end points. Given the critical role of ET-1 in renal function and CKD, alternative approaches are needed to translate ET-1 pathway inhibition to the bedside. With this goal in mind, we sought to better understand *EDN1* gene regulation in light of new findings regarding transcriptional control that continue to emerge from the Encyclopedia of DNA Elements (ENCODE).

Using the University of California-Santa Cruz (UCSC) Genome Browser to interrogate regulatory elements at the *EDN1* locus, we identified a putative promoter downstream of the promoter coding sequence. We hypothesized that this promoter may drive expression of a long non-coding (lnc) RNA. Here we describe a novel lncRNA that is antisense with respect to the ET-1 transcript, *EDN1-AS.* We also identified *EDN1-AS* expression in multiple human cell types including kidney. Using a human kidney proximal tubule cell line (HK-2), we show that CRISPR-mediated deletion of a regulatory element within the *EDN1-AS* promoter resulted in increased levels of *EDN1-AS*. This effect was associated with increased secretion of ET-1 peptide and increased cell proliferation.

## Materials and Methods

### Analysis of EDN1 Chromatin State Using the UCSC Genome Browser

The human *EDN1* chromatin state was analyzed using the UCSC Genome Browser^1^ (Karolchik et al., 2014). The EDN1-AS predicted promoter was identified using the Genome Segments and Broad Chromatin HMM tracks with HUVEC cell information selected. The Transcription Factor ChIP track and DNase Clusters track was also used to analyze transcriptional regulation of the predicted promoter site.

### Cell Culture

HMEC cells were cultured in MEGM^TM^ Mammary Epithelial Cell Growth Medium with BulletKit^TM^ (Lonza) and 10% charcoal stripped FBS. S9 cells were cultured in F12 Ham Kaighn’s modification (F12K) supplemented with 25 mM NaHCO_3_, 4 mM glutamine, 1% Penicillin/Streptomycin and 10% FBS. HK-2 cells were cultured in DMEM/Hamm’s F12 media supplemented with 10% FBS and 1% Penicillin/Streptomycin. HEK293 cells were cultured in DMEM containing 4.5 g/L glucose supplemented with 1% Penicillin/Streptomycin and 10% FBS. All cells were grown in a 37°C incubator, humidified at 5% CO_2_.

### RNA Isolation and DNase Treatment

RNA was isolated from cells using TRIzol (Ambion) per manufacturer instructions. In general, 1 ml TRIzol was used per well in a 6-well plate. Total RNA was treated with DNase (Ambion) per manufacturer instructions to remove genomic DNA. RNA from an adult human female kidney was purchased from Life Technologies.

### Strand-Specific RT-PCR

Human strand-specific *EDN1-AS* primers (SS1-6; Table 1) were designed to lay down at locations progressively closer to the 5′ end of the *EDN1* gene for use in reverse transcriptase reactions. Reverse Transcriptase (RT) from Thermo Fisher was used as per manufacturer instructions. Oligo-dT primers were used as a positive control for any poly-adenylated tailed mRNA. All samples were used in –RT and + RT reactions. Primers have a complementary sequence to the *EDN1* sense strand so they will only anneal to antisense RNA. PCR primers (PCR1 and PCR2) were designed to amplify the same region of cDNA regardless of the strand-specific primer used.

**TABLE 1:** *EDN1-AS* Strand-Specific RT-PCR primers and sequences.

*EDN1-AS* Strand-Specific RT Primers		
Name	Sequence (5′-3′)	
SS1	GCAGTAAAATTATTTTCCTTTATATAACCGGC	
SS2	GTGTCAGCAGTAGATATAATATTTTCATGG	
SS3	CCAAGCTGAAAGGCAAGCCC	
SS4	AACCTATGCTGAGTTCCTCAAGGC	
SS5	CAGAAGAAGTTCAGAGGAACACCTAAG	
SS6	AATAACATTGTCTGGGGCTGGAA	

**PCR Primers**		

**Name**	**Sequence (5′-3′)**	**Product Size**

PCR1	GAAAGAGGTTGGATTGAATTTTGATGTAC	165 bp
PCR2	CAAAGCATGTTCTCAATTTCATTTAGAAATAC	
GAPDH Fwd	AAGAAATGTGCTTTGGGG	166 bp
GAPDH Rev	GACTCCACGACGTACTCA	
EDN1 Fwd	CAGAAGAAGTTCAGAGGAACACC	246 bp
EDN1 Rev	GGTTTGCATTCCAGAGCTTC	

### Dexamethasone Treatment

HK-2 cells were grown to ∼80% confluence in 6-well cell culture plates. To synchronize the circadian clock in all cells, 100 nM Dexamethasone was added for 30 min and then media was changed. After 24 h, cells were trypsinized and RNA was isolated every 2 h for 24 h.

### CRISPR/Cas9 gRNA Design

Guide RNA (gRNA) design, cloning and cell engineering was done as previously outlined (Dükel et al., 2016). Briefly, gRNAs were designed using the online tool CC Top (^2^ Stemmer et al., 2015) using a ∼150 bp sequence of from the predicted human *EDN1-AS* promoter as input. The region was selected based on identified transcription factor binding sites hypothesized to be important to transcription regulation.

### Generation of Recombinant LentiCRISPR Plasmids

gRNA sequences were synthesized as complementary oligonucleotides and included overhangs for insertion into BsmB1-digested LentiCRISPRv2 plasmid (Addgene catalog # 52961). Oligonucleotides were 5′ phosphorylated and annealed as outlined by the Zhang lab^3^ and ligated to linearized LentiCRISPR v2 with Quick Ligase (NEB). Ligation reactions were transformed into Stbl3 bacteria following manufacturer instructions. Using this protocol, four (4) recombinant LentiCRISPR v2 plasmids (designated gRNA 1–4) were cloned and verified by automated Sanger sequencing.

### Lentivirus Generation and Infection of Cells

HEK293 cells were co-transfected with the four LentiCRISPR + gRNA constructs (sgRNA 1, 2, 3, and 4) along with psPAX2 (Addgene #11260) and pMD2.G (Addgene #11259) for generation of pseudotyped lentiviral particles. Media containing virus particles was collected 72 h post-transfection and cleared viral supernatant was then added to HK-2 cells singly and pairwise (e.g., gRNA 1 + 2, 1 + 3, 1 + 4 etc.). 2 μg/ml puromycin was used for 2 weeks to select for cells transduced with virus.

### Isolation of CRISPR/Cas9 KO HK-2 Cells

After puromycin selection, cells were plated into the first of 4 columns of a 96 well plate in 200 μl of media. Cells were then serial diluted into the following columns. After growth time, wells were inspected for the presence of one to three colonies of cells. These wells were then trypsinized with 1 drop of 0.25% Trypsin per well and plated into 10 cm dishes. Cells were allowed to grow undisturbed for 1 week until colony formation. Cloning rings were dipped in sterile vacuum grease to create a seal and pressed down over the colony. Media was aspirated and 1 drop of Trypsin was added. After ∼5 min, trypsinized cells were removed and plated in 6-well plate and allowed to grow. Genomic DNA was isolated from these cells and used for genotyping PCR.

### Sequencing

Gel-purified PCR products from PCR reactions using genotyping primers GT1 and GT2 were cloned into the TA TOPO vector according to the manufacturer’s instructions (Thermo Fisher). Cells with insert were identified by blue/white screening. Plasmids containing inserts were sent to GENEWIZ for sequencing using M13F and M13R primers. The resulting sequences were analyzed with Serial Cloner and sequences from NCBI and UCSC Genome Browser.

### ET-1 ELISA

HK-2 cells were plated into 6-well Transwell plates (Corning) and grown to confluence. Media from HK-2 cell lines was spun down to remove cell debris and then frozen at −80°C until all samples were ready to use. ELISA for ET-1 was performed using the Human Endothelin-1 QuantiGlo ELISA Kit (R&D Systems) according to manufacturer instructions.

### Growth Assay

HK-2 cells were counted and 20,000 cells were plated into 6-well Transwell dishes. Cells were grown under normal conditions for 2, 4, 6, and 8 days. After the specified amount of growth time, cells were treated with Trypsin, diluted, and counted using the BioRad TC20 Automated Cell Counter.

## Results

### Predicted Promoter Region Downstream of the EDN1 Gene

**FIGURE 1:**
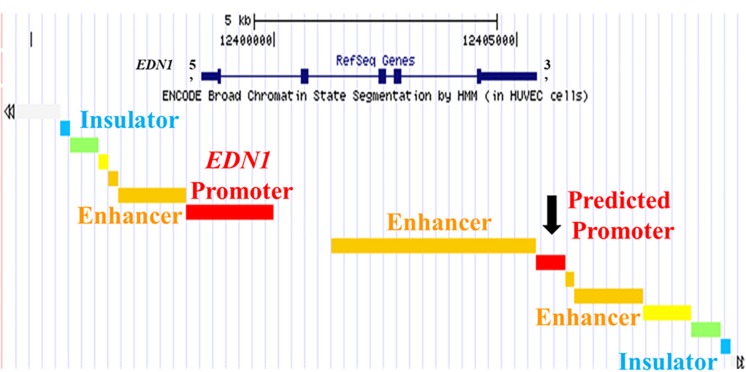
UCSC Genome Browser Predicts Promoter in 3′ UTR of *EDN1* Gene. The University of California Santa Cruz (UCSC) Genome Browser with ENCODE broad chromatin state segmentation by HMM in human umbilical vein endothelial cells (HUVEC) cells shows a predicted promoter (black arrow) in the 3′ untranslated region (UTR) of the *EDN1* gene. The *EDN1* gene is shown at the top (navy blue) with boxes representing exons. The *EDN1* gene position is aligned with the genome segments in the ENCODE broad chromatin state track.

**FIGURE 2:**
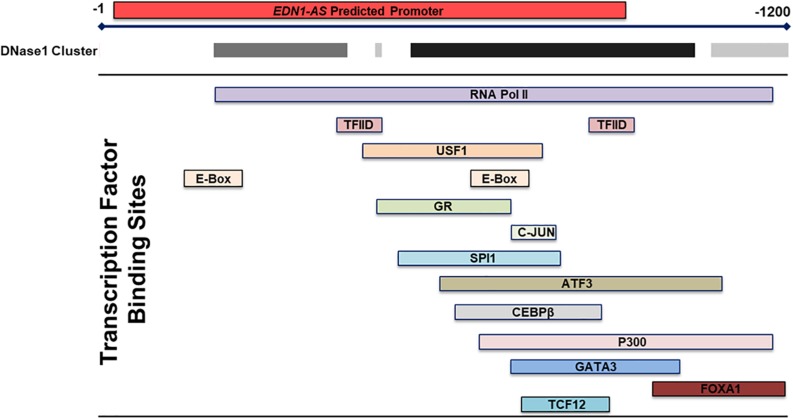
Transcription Factor Binding Sites within the Predicted Promoter in the 3′ UTR of *EDN1*. Using the transcription factor binding sites track within the UCSC Genome Browser, multiple transcription factor binding sites were identified by ChIP within the 3′ UTR *EDN1* predicted promoter. Additionally, the DNase I cluster track showed multiple areas of high DNase sensitivity. The gray box indicates the extent of the hypersensitive region with darker boxes representing a stronger hypersensitivity signal. These data taken together suggests that this predicted promoter region is transcriptionally regulated and that the chromatin is in an open state.

The chromatin state of the human endothlien-1 gene (*EDN1*) gene was analyzed using the UCSC Genome Browser. The Genome Segments track and ChromHMM revealed a predicted promoter region with high confidence immediately downstream of the exon encoding the 3′ untranslated region (UTR) of *EDN1* (about 800 bp long) in human umbilical vein endothelial cells (HUVEC) (Figure 1). This region also contained large clusters of DNase sensitivity sites, suggesting that the chromatin is in an open state and transcription factors could bind to this region (Figure 2). Using the UCSC Genome Browser transcription factor binding site database created using data from chromatin immunoprecipitation (ChIP) assays (ENCODE) (Karolchik et al., 2014), multiple transcription factors including C-JUN, CCAAT/enhancer-binding protein β (CEBPβ), and Forkhead Box A1 (FOXA1) were shown to bind at the location of the predicted promoter region. Additionally, Transcription Factor II D (TFIID), Upstream Transcription Factor 1 (USF1), and Glucocorticoid Receptor (GR) are predicted to bind to this region, and these transcription factors are associated with sites of active transcription. RNA polymerase II was also shown to bind to the predicted promoter region. Based on this information, we hypothesized that the predicted downstream promoter contained a transcription start site and potentially could be a promoter for an antisense RNA.

### EDN1 Anti-sense RNA Detectable in Human Cell Culture Models and Human RNA Samples

**FIGURE 3:**
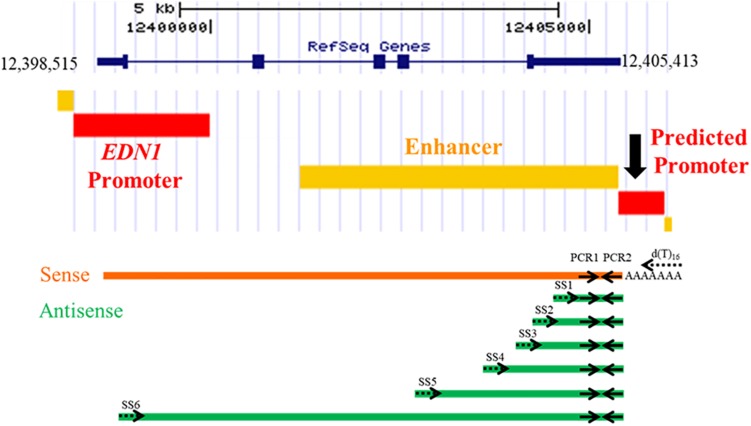
Strand-Specific Reverse Transcriptase PCR Reactions. Strand-specific reverse transcriptase (RT) PCR reactions were designed using primers that would only anneal to an antisense transcript to *EDN1*. A total of 6 different strand-specific primers were used in the RT reactions (SS1–SS6, dotted arrows), which were progressively further away from the predicted 3′ UTR promoter region. For a positive RT control, oligo dT primers were used as it is known *EDN1* mRNA is poly-adenylated. After conversion of RNA into cDNA, PCR1 and PCR2 primers (solid arrows) were used in a PCR reaction to amplify any products generated. PCR1 and PCR2 amplify the same piece of cDNA regardless of the strand-specific primer used, so all bands visualized by gel electrophoresis are the same size (165 bp).

To determine if an antisense RNA is being transcribed from the *EDN1* downstream promoter, a strand-specific reverse transcriptase PCR (ssRT-PCR) strategy was designed (Figure 3). Primers for the reverse transcriptase reactions (RT) were designed to have a complementary sequence to the *EDN1* sense strand, so the primers will only anneal to RNA transcribed from the antisense strand (Table 1). Multiple strand-specific primers were designed at varying distances from the predicted promoter toward the 5′ end of the *EDN1* gene (SS1-6). Oligo dT primers were used as a positive control for the RT reactions. For each RT reaction, a –RT reaction was performed without the reverse transcriptase enzyme as a negative control to ensure the absence of genomic DNA contamination in our samples.

**FIGURE 4:**
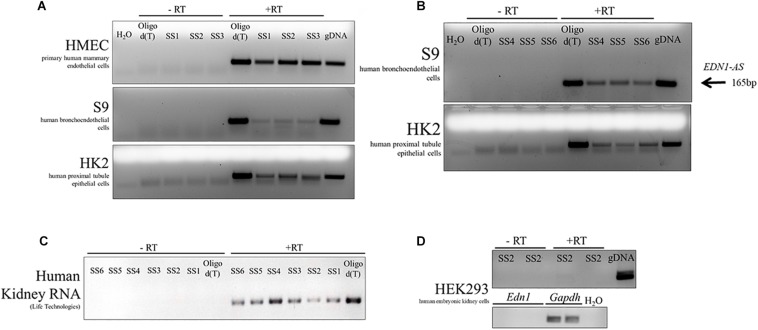
Identification of *EDN1-AS* in Human Cell Culture Models and Human Kidney RNA. Strand-specific reactions were used to test various human cell culture models for the presence of an antisense transcript originating from the predicted promoter region. This transcript has been named *EDN1-AS.* Strand-specific RT primers SS1-3 were used to test for an antisense transcript in primary human mammary endothelial cells (HMEC), human bronchoendothelial cells (S9), and in human proximal tubule epithelial cells (HK2) **(A)**. Oligo dT primers were used as a positive control for the RT reactions. For each PCR reaction, water was used as a negative control and gDNA as a PCR positive control. For each of the strand-specific reactions, a no RT (-RT) control reaction was ran with the same cDNA sample. Strand-specific RT reactions using primers further away from the predicted promoter site (SS4–SS6) were designed to determine if the transcript spanned the entire length of the *EDN1* gene using available samples from S9 and HK2 cells **(B)**. All strand-specific RT primers (SS1–SS6) were used to determine if *EDN1-AS* could be detected in human kidney tissue RNA samples from Life Technologies **(C)**. *EDN1-AS* and *EDN1* transcripts could not be identified in human embryonic kidney cells (HEK293) **(D)**. All gels are representative of at least *n* = 2 per cell line.

Total RNA was isolated from primary human mammary endothelial cells (HMEC), human kidney proximal tubule epithelial cells (HK-2), human bronchoendothelial cells (S9), and human embryonic kidney cells (HEK293). Additionally, human RNA from kidney tissue was obtained from Life Technologies. Each of the RNA samples was treated with DNase and then converted to cDNA using a strand-specific primer or an oligo dT primer. For every sample, both + RT and –RT reactions were performed. The resulting cDNA products were amplified in a PCR using the primers PCR1 and PCR2, which amplified the same region of cDNA regardless of the SS or oligo dT primer used. An antisense RNA transcript was detected in HMEC, HUVEC, HK-2, and S9 cell cultures (Figures 4A,B). The antisense transcript was also detected in human kidney tissue RNA samples (Figure 4C). Interestingly, this antisense RNA was not detected in HEK293 cells, but *EDN1* mRNA was also not detected in these cells (Figure 4D). These results demonstrate that there is an RNA transcribed in an anti-sense direction within the *EDN1* gene locus, and we have designated this transcript *EDN1-AS.* SS primers 4–6 (Figure 3) were designed to detect if the length of *EDN1-AS* spanned the entire *EDN1* coding region (Figures 4B,C). The results indicate that transcription of *EDN1-AS* proceeds at least as far as the first exon of *EDN1*.

### EDN1-AS Exhibits Circadian Rhythm in Expression

**FIGURE 5:**
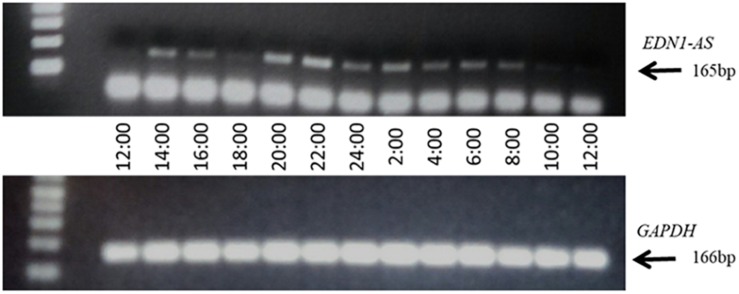
*EDN1-AS* Exhibits a Circadian Rhythm of Expression. HK2 cells were treated with 100 nM dexamethasone for 30 min, after which media was replaced and cells incubated for 24 h. After 24 h (noon), RNA was isolated every 2 h for 24 h total. This RNA was used in strand-specific RT PCR reactions with either SS2 primer (to measure *EDN1-AS*) or random hexamer reactions (to measure *GAPDH*). For the PCR reactions, PCR1 and PCR2 primers were used in *EDN1-AS* reactions and GAPDH Fwd and GAPDH Rev primers were used in the *GAPDH* reactions. Gel representative of *n* = 3 independent experiments.

We and others have shown that the *EDN1* gene is under the regulation of the circadian clock proteins (Hanai et al., 2005; Stow et al., 2012; Kozakai et al., 2014; Richards et al., 2014). Additionally, GR was shown to bind to the *EDN1-AS* promoter region and glucocorticoids have been shown to modulate the circadian clock (Balsalobre et al., 2000; Kino and Chrousos, 2010; Surjit et al., 2011). E-box response elements, to which circadian clock transcription factors bind, were also present in the predicted *EDN1-AS* promoter region. These data suggest that, similarly to the *EDN1* gene, *EDN1-AS* may be under control of the circadian clock and may display a circadian rhythm of expression. To determine if *EDN1-AS* exhibits a circadian rhythm of expression, a 24-h expression profile of the antisense transcript was generated (Figure 5). The HK-2 cells were treated with dexamethasone for 30 min to synchronize their circadian clocks (Feillet et al., 2014). Twenty-four hours after the dexamethasone treatment, RNA was isolated from the HK-2 cells every 2 h for 24 h and the ssRT-PCR protocol was performed as described above. RT reactions with random hexamers were also performed on the same samples in order to detect the levels of *GAPDH* expression as a control. As seen in Figure 5, *EDN1-AS* expression varies over a 24- h period.

### CRISPR Deletion of EDN1-AS Regulatory Region

**FIGURE 6:**
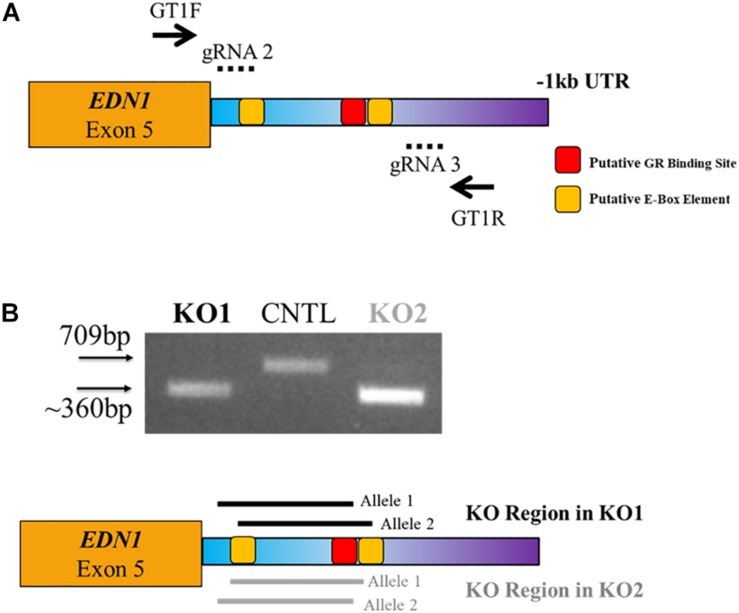
CRISPR/Cas9 Targeted Deletion of GR and E-Box Regulatory Elements within the EDN1-AS Predicted Promoter Region. CRISPR/Cas9 technology was utilized to create biallelic KO of a portion of the predicted Edn1AS promoter region, which contained GR and E-Box regulatory sites. Four guide RNAs (gRNAs) were designed to create deletion of this region **(A)**. LentiCRISPR was used to deliver the gRNAs to HK2 cells. Puromycin was used to screen cells. Using genotyping primers, GT1F and GT1R (solid arrows), two clones were identified that had a KO of approximately 350 bp generated with gRNAs 2 and 3 (dotted lines) **(B)**. Sequencing results reviled KO1 has a biallelic KO of 297 bp with 64 bp additional KO 5′ of this region on one allele and 78 bp KO 3′ of this region on the other allele (black solid lines). KO2 has a biallelic KO of 310 bp with 46 bp additional KO 3′ of this region on one allele and 71 bp KO 5′ of this region on the other allele (gray solid lines). Both KOs have a deletion of the 1st E-Box response element and the GR element. In addition, we identified control (CNTL) cells that had undergone the CRISPR process, but retained WT copes of the *EDN1-AS* regulatory region.

Since the expression of *EDN1-AS* seems to be under circadian regulation, we wanted to investigate the effect of removing the E-box response element and GR binding site in the predicted promoter region of *EDN1-AS.* In order to create a KO of this regulatory region in HK-2 cells, we utilized CRISPR/Cas9 technology. Guide RNAs (gRNAs) were designed using the online tool CC Top (Stemmer et al., 2015; Table 2). gRNAs were selected based on location and predicted off-target sites. If possible, only gRNAs with no off-target sites or those with sites only in regions between genes were chosen. No gRNAs were used that had predicted off-target sites in exonic or intronic regions of any gene to minimize possible off-target effects. Various combinations of all the gRNAs were used in order to create a biallelic KO of the *EDN1-AS* regulatory region. The gRNA combination of gRNA 2 and 3 resulted in two HK-2 cell lines that had a homozygous deletion of an approximately 350 bp region within the predicted *EDN1-AS* promoter by genotyping PCR (Figure 6). Sanger sequencing using genomic DNA from these cell lines was used to confirm the KO region. In addition to the two homozygous KO cell lines, control (CNTL) cell lines that had undergone the CRISPR process, but did not have KO of the *EDN1-AS* regulatory region, were identified to use as control cells for future experiments.

**TABLE 2:** Guide RNAs for CRISPR/Cas9 and genotyping primers.

gRNAs		
Name	Sequence (5′-3′)	
hEDN1AS CRISPR 1F	CACCGATAGAAAAGTGACAATTAGA	
hEDN1AS CRISPR 1R	AAACTCTAATTGTCACTTTTCTATC	
hEDN1AS CRISPR 2F	CACCGTTGATTTTATACTGCATTG	
hEDN1AS CRISPR 2R	AAACCAATGCAGTATAAAATCAAC	
hEDN1AS CRISPR 3F	CACCGGCCTGCTCAGAGCAAGTAG	
hEDN1AS CRISPR 3R	AAACCTACTTGCTCTGAGCAGGCC	
hEDN1AS CRISPR 4F	CACCGCCTGGTGTCTCTACCTCCAT	
hEDN1AS CRISPR 4R	AAACATGGAGGTAGAGACACCAGGC	

**Genotyping Primers**		

**Name**	**Sequence (5′-3′)**	**PCR Product**

GT1F	CATGCTTTGTTTTGCCTGTCA	709 bp
GT2R	AGGCAAAGTGGAGAGGAAGT	

### KO of EDN1-AS Regulatory Region Results in Increased EDN1-AS and EDN1 Expression

**FIGURE 7:**
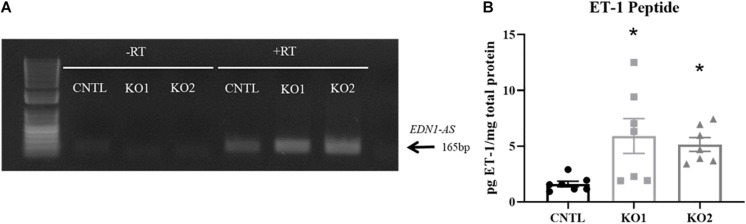
CRISPR/Cas9 KO of *EDN1-AS* Regulatory Elements Results in Increased *EDN1-AS* and *EDN1* Expression. *EDN1-AS* expression levels were examined in CNTL and CRISPR/Cas9 KO cells by strand-specific RT PCR. For each reaction, 20 ng of cDNA was used in PCR reactions to amplify *EDN1-AS*. CRISPR/Cas9 KO cells had more *EDN1-AS* expression visualized by gel electrophoresis **(A)** (gel representative of *n* = 3 for CNTL and *n* = 2 for each KO cell line). To determine if these differences in *EDN1-AS* expression had an effect on *EDN1* expression, the product of the *EDN1* gene, endothelin-1 (ET-1) peptide was measured. Cells were placed in Transwells dishes and grown to confluence. Media from these cells was used in an endothelin-1 ELISA (R&D QuantiGlo ET-1 ELISA) to determine the amount of secreted ET-1 relativized to total protein concentration **(B)**. One-way ANOVA was used to determine significant differences between each KO and the CNTL cell line, *n* = 7 for each cell line. *P < 0.05.

To determine how the KO of the *EDN1-AS* regulatory region containing the predicted GR and circadian clock binding sites affected the expression of *EDN1-AS*, we performed our ssRT-PCR protocol on the KO cell lines in addition to the CNTL cells. The CRISPR KO cells had higher expression of *EDN1-AS* compared to CNTL cells (Figure 7A). Secreted endothelin-1 peptide (ET-1) was measured to determine if the increase in expression of the *EDN1-AS* gene led to an increase in ET-1 peptide level. Briefly, KO and CNTL cells were plated into Transwell plates and media was collected from confluent cells. The media was then analyzed via an ET-1 ELISA to measure total ET-1 peptide levels. ET-1 peptide levels were normalized to total protein concentration measured by BCA assay. The CRISPR KO cells had a significant increase in total secreted ET-1 peptide levels compared to the CNTL cells (Figure 7B).

### Increased ET-1 Production Associated With Increased Growth Rate

**FIGURE 8:**
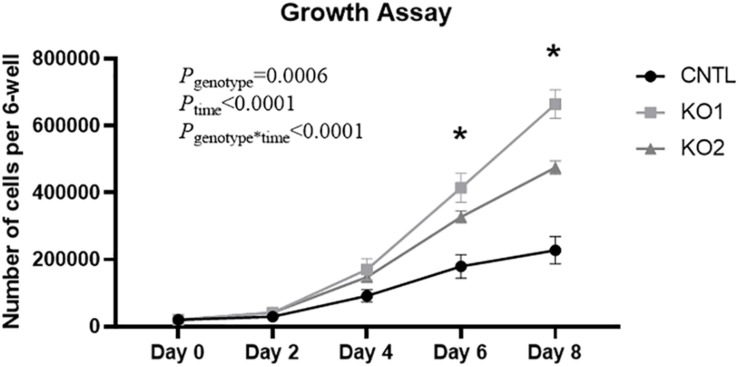
KO of *EDN1-AS* Regulatory Elements Results in Increased Growth Rate of HK2 Proximal Tubule Cells. Because high expression of ET-1 is known to lead to cell proliferation in kidney proximal tubule cells, growth rate of CNTL and CRISPR/Cas9 KO cells was measured. 20,000 cells were plated into a 6-well dish and allowed to grow under normal conditions for 2, 4, 6, or 8 days. An automated cell counter was used to determine the total amount of cells in each well. Two-way ANOVA with repeated measures was used to determine genotype differences. ^∗^*P* < 0.05 by *T-*test within 1 day denotes that both KO cell lines are significantly different from the CNTL cell line, *n* = 4.

High expression of ET-1 in human proximal tubule cells, like HK-2 cells, is associated with increased cell proliferation (Zoja et al., 1995; Seccia et al., 2016). To determine if the increased ET-1 levels caused by KO of the *EDN1-AS* regulatory region affected cell proliferation, growth rates of the KO and CNTL cells were measured. Briefly, 20,000 cells were plated into 6-well dishes and counted after 2, 4, 6, and 8 days of growth. Both CRISPR KO cell lines grew significantly faster than the CNTL cells (Figure 8). Significant differences between individual KO cells and the CNTL cells total cell count were detected on day 6 and day 8.

## Discussion

Here we report the identification of a novel lncRNA, *EDN1*-AS, that is antisense to the ET-1 coding sequence. *EDN1-AS* is expressed in human mammary epithelial cells, human bronchoendothelial cells, human kidney proximal tubule cells, and in human kidney. Using a strand-specific RT-PCR approach, we demonstrated that *EDN1-AS* comprises the entire length of the *EDN1* sense coding sequence. *EDN1-AS* appears to exhibit rhythmic expression in HK-2 cells. CRISPR-mediated deletion of regulatory sequence upstream of *EDN1-AS* containing a GR element and an E-box resulted in increased expression of *EDN1-AS* in HK-2 cells. This effect was associated with increased ET-1 peptide secretion from the cells and increased growth rates.

CRISPR-mediated knockout of the region containing the predicted GR element and E-box resulted in increased expression of *EDN1-AS*, suggesting that the deleted regulatory region contains a repressive element. Given that GR elements can be bound by both GR and MR and these transcription factors can mediate positive and negative effects on gene regulation (Koning et al., 2019), this result is not surprising. Deletion of a GR element in the promoter of αENaC revealed a role for repressive regulation (Lin et al., 1999). Likewise, circadian clock proteins such as PER1 interact with E-box elements to mediate positive and negative effects on gene expression (Cox and Takahashi, 2019). Promoter analysis of the gonadotropin-releasing hormone demonstrated a key role for the E-box elements and a repressive role for PER1 (Resuehr et al., 2007). The concept that PER1 may mediate negative regulation of *EDN1-AS* is consistent with our previous observations that PER1 in the kidney mediates repression of the sense *EDN1* mRNA (Stow et al., 2012; Richards et al., 2014).

The non-coding RNA field has exploded in recent years due to the wealth of information provided by ENCODE. It is now estimated that more than 50% of the human genome is transcribed but only 1.2% of the genome encodes proteins (Ransohoff et al., 2018). Non-coding RNAs thus comprise a significant portion of the transcriptome, but our understanding of these RNA species is in its infancy. Some lncRNAs are natural antisense transcripts (NAT) because they arise by transcription of the strand opposite an mRNA (Khorkova et al., 2014). Our data suggest that *EDN1-AS* is a NAT with functional effects on ET-1 action. We estimate that the length of unprocessed *EDN1-AS* is at least 5 kb, based on the signal detected in HK2 and S9 cells and human kidney using SS6, which is located in the first exon of the sense *EDN1* sequence (Figure 3). The ssRT-PCR results in Figure 4 demonstrate that *EDN1-AS* transcripts spanning the length of the *EDN1* gene are present in human kidney and bronchoendothelial cells. Whether or not *EDN1-AS* is subject to splicing or other post-transcriptional regulation remains to be determined.

Our report is the first description of an antisense lncRNA associated with the *EDN1* locus. According to LNCipedia, four lncRNAs have been identified near the *EDN1* gene (Volders et al., 2019). However, all four of these are associated with the sense strand. Functional relevance of these lncRNAs has not been described. Interestingly, a SNP in PHACTR, a gene ∼400,000 bp downstream of *EDN1*, has been associated with regulation of ET-1 levels (Gupta et al., 2017). This region appears to interact with an area of open chromatin located in a “gene desert” between the *EDN1* and *PHACTR* loci. These results, together with our current report, strongly suggest a new level of complexity in the regulation of *EDN1*.

A limitation of the current study is that the mechanism by which *EDN1-AS* affects ET-1 expression remains unclear. Many antisense RNAs act in an inhibitory manner, however, as the knowledge base regarding non-coding RNA increases, it is becoming increasingly clear that NATs act through myriad mechanisms (Wanowska et al., 2018). One example of a NAT that exerts positive effects on target mRNA expression is PTENP1, an antisense transcribed from the locus of the tumor suppressor gene PTEN. PTENP1 acts as a miRNA sponge for miR21, thus protecting the PTEN transcript from miRNA-mediated degradation (Wang et al., 2016). Another example of positive regulation by a NAT is BACE1-AS, which is transcribed from the β-amyloid cleaving enzyme 1 (BACE1) locus. BACE1-AS interacts with the BACE1 sense mRNA, masking a miRNA binding site and thus protecting BACE1 from miRNA-mediated degradation (Faghihi et al., 2010). BACE1-AS shows promise as a potential therapeutic in Alzheimer’s disease (Ge et al., 2020). Given that ET-1 is subject to regulation by miRNA (Jacobs et al., 2013), it is tempting to speculate that *EDN1-AS* may function as a positive regulator of *EDN1* expression through a miRNA-protection mechanism. Future experiments are needed to test this hypothesis.

The lncRNAs are a newly recognized mechanism for gene regulation and are being explored as potential therapeutic targets in a variety of diseases (Li et al., 2018; Pecero et al., 2019; Wang et al., 2020), including diabetic nephropathy (Ge et al., 2019; Yang et al., 2019). A nucleic acid-based approach could allow greater specificity than pharmacological compounds, reducing side effects and allowing tissue-specific delivery (Sharma and Watts, 2015; Ku et al., 2016). Future studies are needed to determine whether or not manipulation of *EDN1-AS* could have therapeutic benefits. Although expression of *EDN1-AS* was confirmed in a variety of cell types and even in human kidney in the present study, the functional studies related to *EDN1-AS* are limited to HK-2 cells. In HK-2 cells, increased expression of *EDN1-AS* was associated with increased cell proliferation, which may have implications for manipulating *EDN1-AS* levels in a CKD setting.

## Data Availability Statement

The datasets generated for this study are available on request to the corresponding author.

## Author Contributions

LD, KS, LJ, CW, BC, KB, and MG conceived and designed research. LD, KS, SM, DB, SB, LJ, KA, RP, and KB performed experiments. LD, KS, SM, DB, and MG analyzed data. LD and MG interpreted results of experiments, prepared figures, and drafted manuscript. LD, SM, DB, SB, LJ, KB, BC, and MG edited and revised manuscript. LD, KS, SM, DB, SB, LJ, KA, RP, CW, KB, BC, and MG approved final version of manuscript.

## Conflict of Interest

The authors declare that the research was conducted in the absence of any commercial or financial relationships that could be construed as a potential conflict of interest.

## Footnotes

1 http://genome.ucsc.edu

2 crispr.cos.uni-heidelberg.de

3 https://www.addgene.org/crispr/zhang
